# IKKα Contributes to Canonical NF-κB Activation Downstream of Nod1-Mediated Peptidoglycan Recognition

**DOI:** 10.1371/journal.pone.0015371

**Published:** 2010-10-15

**Authors:** Man Lyang Kim, Hyun Gyeong Jeong, Christoph Alexander Kasper, Cécile Arrieumerlou

**Affiliations:** Biozentrum, University of Basel, Basel, Switzerland; Charité-University Medicine Berlin, Germany

## Abstract

**Background:**

During pathogen infection, innate immunity is initiated via the recognition of microbial products by pattern recognition receptors and the subsequent activation of transcription factors that upregulate proinflammatory genes. By controlling the expression of cytokines, chemokines, anti-bacterial peptides and adhesion molecules, the transcription factor nuclear factor-kappa B (NF-κB) has a central function in this process. In a typical model of NF-κB activation, the recognition of pathogen associated molecules triggers the canonical NF-κB pathway that depends on the phosphorylation of Inhibitor of NF-κB (IκB) by the catalytic subunit IκB kinase β (IKKβ), its degradation and the nuclear translocation of NF-κB dimers.

**Methodology:**

Here, we performed an RNA interference (RNAi) screen on *Shigella flexneri*-induced NF-κB activation to identify new factors involved in the regulation of NF-κB following infection of epithelial cells by invasive bacteria. By targeting a subset of the human signaling proteome, we found that the catalytic subunit IKKα is also required for complete NF-κB activation during infection. Depletion of IKKα by RNAi strongly reduces the nuclear translocation of NF-κB p65 during *S. flexneri* infection as well as the expression of the proinflammatory chemokine interleukin-8. Similar to IKKβ, IKKα contributes to the phosphorylation of IκBα on serines 32 and 36, and to its degradation. Experiments performed with the synthetic Nod1 ligand L-Ala-D-γ-Glu-meso-diaminopimelic acid confirmed that IKKα is involved in NF-κB activation triggered downstream of Nod1-mediated peptidoglycan recognition.

**Conclusions:**

Taken together, these results demonstrate the unexpected role of IKKα in the canonical NF-κB pathway triggered by peptidoglycan recognition during bacterial infection. In addition, they suggest that IKKα may be an important drug target for the development of treatments that aim at limiting inflammation in bacterial infection.

## Introduction

During pathogen infection, structurally conserved microbial molecules are recognized by germline-encoded pathogen recognition receptors (PRRs) that function as sensors for non-self detection and initiate innate immunity [1], [2]. PRRs include transmembrane proteins such as Toll-like receptors and C-type lectin receptors, as well as cytoplasmic proteins such as retinoic acid-inducible gene (RIG)-I-like receptors and NOD-like receptors [3], [4], [5]. They are expressed in macrophages and dendritic cells but also in various non-professional immune cells including epithelial and endothelial cells. PRRs recognize a large variety of pathogen associated molecular patterns (PAMPs) from both extracellular and intracellular pathogens including lipopolysaccharide, peptidoglycan, lipoproteins, dsRNA, ssRNA, CpG-DNA and flagellin [6]. Signaling pathways of PAMP recognition converge into the activation of the pleiotropic transcription factor nuclear factor-kappa B (NF-κB) that, in the context of innate immunity, regulates the expression of proinflammatory genes encoding cytokines, chemokines, anti-bacterial peptides and adhesion molecules [7]. The mammalian NF-κB family consists of the members RelA/p65, RelB, c-Rel, p50 (NF-κB1) and p52 (NF-κB2) [8]. All five proteins share a Rel homology domain and form homo- and heterodimers that regulate transcription by binding to κB sites in promoters or enhancers of target genes. In unstimulated cells, most of the NF-κB dimers are sequestrated in the cytoplasm by the proteins of the Inhibitor of NF-κB (IκB) family whose prototype is IκBα. In the canonical pathway of NF-κB activation triggered by most stimuli including bacterial and viral infection, cytokines and stress-induced responses, phosphorylation of IκBα on Serine 32 and Serine 36 residues by the IκB kinase (IKK) complex is a decisive regulatory step [9]. The IKK complex is comprised of three subunits: two catalytic subunits, IKKα and IKKβ, and the regulatory scaffold component NF-κB essential modulator (NEMO). The respective contribution of IKKα and IKKβ in the phosphorylation of IκBα is unclear. Although it is generally accepted that IKKβ is critical for IκBα phosphorylation through the canonical pathway, two recent reports demonstrate the equal importance of IKKα for the activation of NF-κB by the inflammatory cytokines interleukin-1 (IL-1) in mouse embryonic fibroblasts and tumor necrosis factor alpha (TNFα) in HeLa cells [10], [11]. The phosphorylation of IκBα is followed by its rapid polyubiquitination and subsequent degradation by the 26S proteasome complex [12]. The release of NF-κB with unmasked nuclear localization sequence leads then to the translocation of the transcription factor to the nucleus where it regulates gene expression [13].

Although the role of NF-κB is central to many pathways triggered by pathogen recognition, the molecular processes that govern its activation are only partially elucidated. In particular, the mechanisms triggered by the detection of invasive bacteria such as the pathogen *Shigella flexneri* remain largely uncharacterized. *S. flexneri* makes use of a type III secretion (T3S) apparatus to locally rearrange the host actin cytoskeleton and penetrate into intestinal epithelial cells [14]. Once internalized, bacteria multiply in the host cytoplasm and use actin-based motility to spread to adjacent epithelial cells. During infection, massive inflammation is observed in colonic mucosal tissues [15]. In infected epithelial cells, intracellular bacteria release peptidoglycan-derived peptides that are specifically recognized by Nod1 [16]. Upon ligand binding, Nod1 homo-dimerizes and recruits the downstream kinase RICK/RIPK2 through heterologous caspase-recruitment domain interactions [17]. This converges to the sequential recruitment and activation of the TAK1/TAB1/TAB2 and IKKα/IKKβ/IKKγ complexes, the nuclear translocation of NF-κB and the upregulation of proinflammatory genes encoding for cytokines and chemokines, including interleukin-8 (IL-8) and TNFα [18]. The chemokine IL-8 recruits polymorphonuclear cells to the site of infection and therefore contributes to contain the dissemination of bacteria within the intestinal tissue. Interestingly, *S. flexneri* uses the T3S apparatus to secrete several effectors that alter multiple signaling pathways in infected cells and reduce the expression of proinflammatory genes [19]. Among others, the effector OspF suppresses the expression of IL-8 by dephosphorylating the MAP kinases p38 and ERK in the nucleus of infected cells [20], [21], thereby impairing the phosphorylation of Histone H3, a process that regulates the access of chromatin to transcription factors.

Here, we performed an RNA interference (RNAi) screen on *S. flexneri*-induced NF-κB activation to identify new factors involved in the regulation of NF-κB following infection of epithelial cells by invasive bacteria. By targeting a subset of the human signaling proteome, we identified IKKα as a protein required for *S. flexneri*-induced NF-κB nuclear translocation and IL-8 secretion in HeLa cells. This result was unexpected because, except for IL-1 and TNFα [10], [11], it is generally accepted that IKKβ is the component of the IKK complex involved in the canonical pathway of NF-κB activation. Depletion of IKKα or IKKβ indicated that *S. flexneri*-induced NF-κB activation in HeLa cells requires indeed both catalytic subunits. We further characterized the role of IKKα and found that, during *S. flexneri* infection, IKKα was required for the phosphorylation of IκBα on serines 32 and 36, and for its degradation. Experiments performed with the synthetic Nod1 ligand L-Ala-D-γ-Glu-meso-diaminopimelic acid (Tri-DAP) indicated that IKKα was involved in Nod1-mediated signaling pathway of NF-κB activation. Taken together, these results show that, although Nod1 signaling triggers the canonical pathway of NF-κB activation, both IKKα and IKKβ are required for full NF-κB activation.

## Materials and Methods

### Antibodies and reagents

Antibodies against NF-κB p65, IκBα and IKKα were obtained from Santa Cruz Biotechnology (Santa Cruz, USA) whereas the anti-actin was from Chemicon (Billerica, USA) and the anti-phospho-IκBα was from Cell signaling technology (Beverly, USA). The anti-mouse IgG-Cy5 was obtained from Zymed (San Francisco, USA) and the anti-rabbit IgG-HRP and anti-mouse IgG-HRP from GE Healthcare (Pittsburgh, USA). Hoechst and FITC-phalloidin were from Invitrogen (Carlsbad, USA), TNFα from R & D systems (Minneapolis, USA).

### Cell culture and transfection

HeLa cells were maintained in Dulbecco's modified Eagle's medium (high glucose) supplemented with 10% fetal bovine serum, 100 units/ml penicillin, and 100 µg/ml streptomycin at 37°C in 10% CO_2_. HeLa cells were transfected with siRNAs and DNA plasmids using Lipofectamine 2000 (Invitrogen, Carlsbad, USA) and jetPEI (Poly plus transfection, Illkirch, France), respectively. siRNAs ON-TARGETplus SMARTpool targeting IKKα (#L-003473-00-005) and ON-TARGETplus siCONTROL (Dharmacon, Dallas, USA) were used in all our study except for the experiments where IKKα, IKKβ and NEMO were silenced in parallel. In this case, all siRNAs were from Qiagen (Valencia, CA, USA).

### *In vitro* diced siRNA library

An *in vitro* diced siRNA library targeting 132 genes coding for a subset of the signaling proteome was generated as previously described [22], [23], [24]. Briefly, for each gene, a 600 base pair cDNA was generated by PCR from a total cDNA library. An additional set of nested primers was used to add T7 promoters at both ends of the final cDNA fragment. Nested PCR products were subject to *in vitro* transcription, dicing, and purification to produce gene specific siRNA pools. Dicing was performed with the turbo dicer siRNA generation kit from Genlantis (San Diego, USA). The concentration of all siRNA pools was normalized.

### Bacterial strains

The *S. flexneri* strains M90T wild-type and the *icsA* (*virG*) deletion mutant (Δ*virG*) were generously provided by Dr. P. Sansonetti (Institut Pasteur, Paris, France). All strains were transformed with the pMW211 plasmid to express the DsRed protein under control of a constitutive promoter. The pMW211 plasmid was a generous gift from Dr. D. Bumann (Biozentrum, University of Basel, Switzerland). The Δ*ospF* deletion mutant used in IL-8 expression experiments, was generated from the Δ*virG* mutant by allelic exchange using a modification of the lambda red-mediated gene deletion [25]. Briefly, the genes for lambda red recombination were expressed from the pKM208 plasmid [26]. The chloramphenicol resistance cassette (cat) of the pKD3 plasmid was amplified using the primers listed in Table 1. After DpnI digestion, the PCR product was electroporated into the Δ*virG* mutant. Recombinants were selected on TSB plates containing 5 or 10 µg ml^−1^ chloramphenicol. The cat cassette was removed by transformation of pCP20 and incubation at 30°C on TSB plates containing 100 µg ml^−1^ ampicillin [25]. Single colonies were screened by PCR.

**Table 1:** Oligonucleotide primers used to generate the Δ*ospF* mutant.

Mutant	Forward	Reverse
Δ*ospF*	ATTCTATTATATAGATAAAATATCTCCTGCAAAAGATACGGGTATTTTTGTGTAGGCTGGAGCTGCTTCG	TCAAAAGTTCGATGTTCCACCACATCGACCGTAGAAGAGATGAGATAGTACATATGAATATCCTCCTTAG

### Infection assay

Bacteria were routinely grown in tryptic soy broth (TSB) medium, used in exponential growth phase, and treated with poly-L-lysine prior infection. HeLa cells, seeded in 96-well plates, were serum starved for 30 min and infected with *S. flexneri* at a multiplicity of infection (MOI) of 10. Immediately after adding bacteria, the plates were centrifuged for 5 min at 2000 rpm and placed at 37°C for 30 min. Extracellular bacteria were killed by addition of gentamycin (50 µg/ml).

### Immunofluorescence

Cells were fixed with 4% PFA for 6 min and permeabilized in 0.5% Triton X-100 for 10 min. They were, then, incubated with a mouse monoclonal anti-p65 antibody (1 µg/ml) overnight at 4°C and stained with a Cy5-conjugated secondary antibody and Hoechst (10 µg/ml) for 40 min at room temperature.

### siRNA screen of *S. flexneri*-induced p65 nuclear translocation

The *in vitro* diced siRNA library was screened on *S. flexneri*-induced p65 translocation assay in a 96-well format. The firefly *luciferase* (GL3) siRNA was used as a non-silencing negative control as described previously [22], [23], [24]. siRNA pools against Nod1, RIPK2 and Src were used as positive controls. The screen was performed three times in duplicate as follows. Three thousand HeLa cells per well were transfected by reverse transfection with the individual 132 siRNA pools in 96-well plates. After 48 hours, cells were infected with DsRed *S. flexneri* at MOI of 10 for 90 min and then fixed, permeabilized, and stained for p65, F-actin, and DNA. Images were acquired at 12 random sites of each well using the automated ImageXpress microscope (Molecular devices, Sunnyvale, USA). At each site, images at 360 nm, 480 nm, 594 nm, 640 nm were acquired to visualize Hoechst, Phalloidin, DsRed *S. flexneri* and p65, respectively. The nuclear localization of p65 was automatically quantified by using the Enhanced-Translocation module of MetaXpress (Molecular devices, Sunnyvale, USA). Briefly, the Hoechst staining was used as a mask to automatically identify nuclei in the p65 staining image. The cytoplasmic area was defined by a ring around each nucleus. For each cell, the ratio of p65 intensity in the nucleus and in the cytoplasmic ring defined as the Nuc/Cyt p65 ratio was calculated and averaged over several thousands of cells per well. The results of the screen were expressed as individual scores. The score of a particular gene represents the fold standard deviation from the mean of the GL3 control wells. A negative or a positive sign was assigned to the score when the Nuc/Cyt p65 ratio was lower or higher than the GL3 control ratio, respectively.

### Enzyme- linked Immunosorbent Assay (ELISA)

IL-8 secretion was measured by ELISA in the supernatant of HeLa cells 6 hours post infection. Cell-free supernatants from triplicate wells were analyzed for their IL-8 content using a commercial ELISA kit (BD Pharmingen, San Jose, USA).

### Western blot analysis

HeLa cells were transfected with siRNAs in a 6-well plate. 72 hours post transfection, cells were lysed in Phosphosafe Extraction Buffer (Novagen, Darmstadt, Germany) supplemented with 1x protease inhibitor cocktail (Calbiochem, Darmstadt, Germany). Protein concentration was measured using the bicinchonic acid (BCA) kit (Pierce, Rockford, USA). Equal amounts of proteins were resolved by SDS-PAGE and transferred to Hybond C-Extra membrane (Amersham Bioscience, Pittsburgh, USA) for immunoblotting with individual antibodies. Primary antibodies were detected using horseradish peroxidase-conjugated anti-rabbit or anti-mouse IgG antibodies, and visualized with the ECL system (Pierce). Quantification of the blots was performed using the densitometry feature of Photoshop.

### Tri-DAP treatment

Cells were serum starved 30 min before treatment with L-Ala-γ-D-Glu-mesoDAP (Tri-DAP). Tri-DAP treatment was performed by calcium phosphate transfection using 0.4 µg/ml Tri-DAP final concentration for 90 min. Then cells were fixed with 4% PFA and analyzed by immunofluorescence as described above.

### Statistical analysis

Data are expressed as mean ± standard deviation calculated from the number of replicates specified in figure legends. p values were calculated with a two-tailed two-sample equal variance t-test.

## Results

### RNAi screen identifies the role of IKKα in the nuclear translocation of NF-κB p65 during infection of epithelial cells by *S. flexneri*

**Figure 1:**
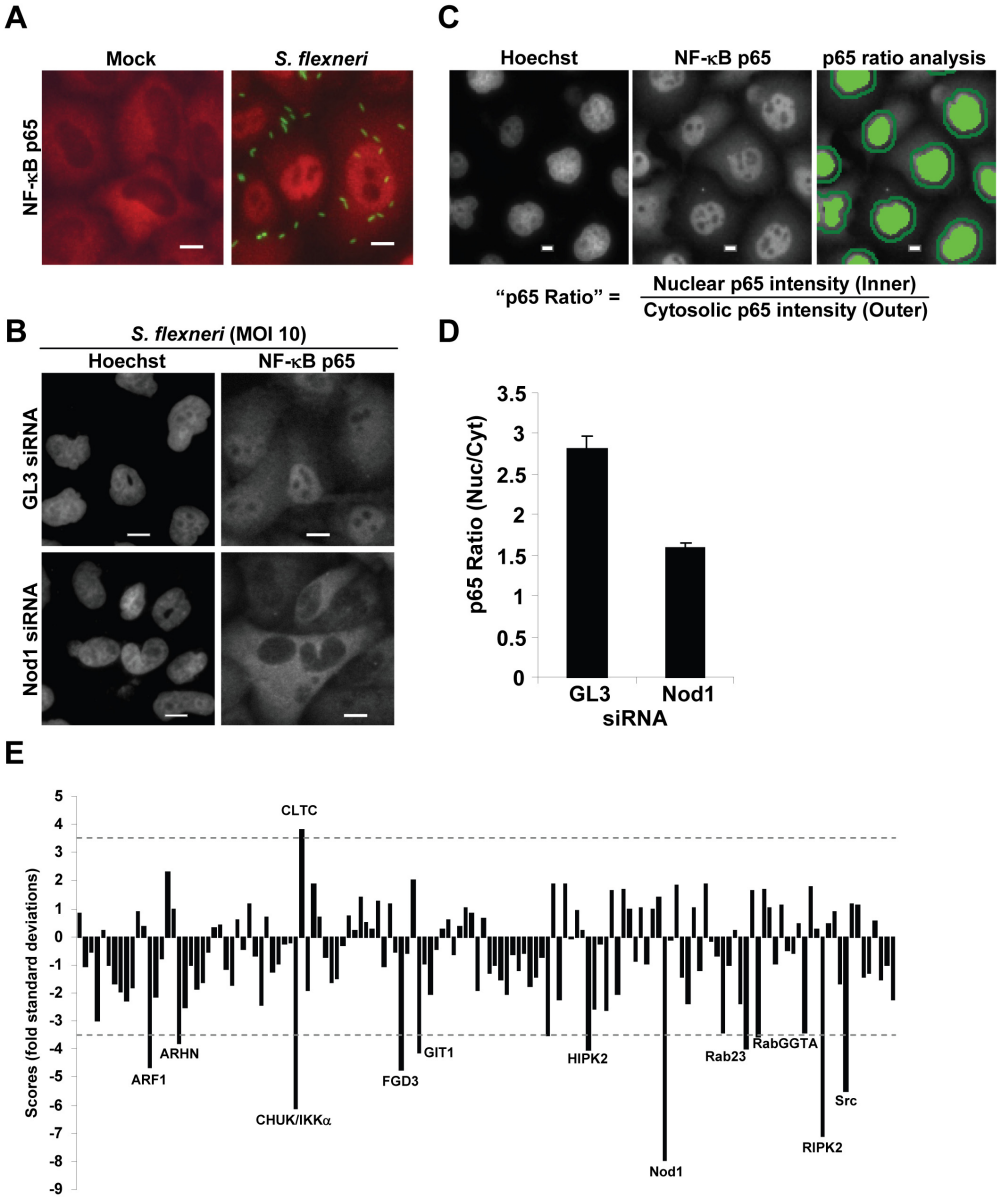
RNAi screen identifies the role of IKKα in p65 nuclear translocation during *S. flexneri* infection. (**A**) NF-κB p65 translocates to the nucleus in response to *S. flexneri* infection. HeLa cells were left untreated (Mock) or infected with dsRed-expressing *S. flexneri* for 60 minutes at MOI = 10. NF-κB p65 localization was visualized by immunofluorescence microscopy. An overlay image is shown for *S. flexneri* (green) and p65 (red). (**B**) Depletion of Nod1 by RNAi inhibits the nuclear translocation of NF-κB p65 in response to *S. flexneri* infection. HeLa cells were transfected with GL3 or Nod1 siRNA and infected with *S. flexneri* for 60 minutes at MOI = 10. p65 and DNA were visualized by anti-p65 and Hoechst staining, respectively. (**C**) Images illustrating the quantification of the p65 ratio. The Hoechst staining was used as a mask to automatically identify the nuclei in the p65 staining image. The cytoplasmic area was defined by a ring surrounding each nucleus. Scale bars, 10 µm (**D**) Quantification of the p65 ratio in control and Nod1-depleted cells following infection by *S. flexneri*. Results represent the mean ± SD of 12 images; graph representative of 3 independent experiments. (**E**) RNAi screen of *S. flexneri*-induced p65 nuclear translocation in HeLa cells. Scores are fold standard deviations from the mean of GL3 control wells. Dashed lines represent scores of ±3.5 (See Materials and Methods for details).

Over the past 10 years, RNAi has been an essential tool to study gene function in mammalian cells [24], [27], [28]. Using *S. flexneri*-induced NF-κB activation in HeLa cells as a model system, we performed an image-based RNAi screen to identify new proteins involved in the molecular mechanisms that control NF-κB activation following pathogen recognition. The nuclear localization of NF-κB p65 visualized by anti-p65 immunofluorescence microscopy was used as readout for NF-κB activation. In uninfected cells, p65 was mainly localized in the cytoplasm (Figure 1A, left panel). In contrast, following infection with dsRed-expressing *S. flexneri*, a strong nuclear translocation of p65 was observed (Figure 1A, right panel). To validate the RNAi approach, silencing of the intracellular pattern recognition receptor Nod1 involved in *S. flexneri* recognition was tested. HeLa cells were transfected with pools of *in vitro* diced small interference RNA (siRNA) targeting Nod1 or the firefly *Luciferase* used as control (GL3). After 48 hours, cells were infected with *S. flexneri*, stained for p65 and DNA with an anti-p65 antibody and Hoechst, respectively. Visual inspection of images showed that *S. flexneri*-induced p65 nuclear translocation was suppressed in Nod1-depleted cells (Figure 1B). This observation was quantified by measuring for each cell the ratio of p65 intensity in the nucleus and in the cytoplasm by automated image processing (Figure 1C). The Hoechst staining was used to automatically identify nuclei whereas the cytoplasmic area was defined by a ring around each nucleus. Quantification of the Nucleus/Cytoplasm p65 intensity ratio (Nuc/Cyt p65 ratio) confirmed that the depletion of Nod1 inhibited the nuclear translocation of p65 induced during infection (Figure 1D), and therefore, that the RNAi approach was suitable to identify new proteins involved in the activation of NF-κB during *S. flexneri* infection of epithelial cells.

An *in vitro* diced siRNA library targeting 132 genes from the human signaling proteome was screened on *S. flexneri*-induced p65 nuclear translocation as described in Materials and Methods. For each gene, a score representing the fold standard deviation from the mean of GL3 control wells was calculated (Figures 1E and Table S1). A negative or a positive sign was attributed to the score when the Nuc/Cyt p65 ratio was lower or higher than the GL3 ratio, respectively. As expected, Nod1 and RIPK2, two key proteins involved in *S. flexneri*-induced NF-κB activation [16], [29], as well as Src, a tyrosine kinase required for bacterial entry into cells [30], obtained strong negative scores (Figures 1E and Table S1). Using an arbitrarily determined cut off score value of +/− 3.5, the proteins CHUK/IKKα, FGD3, Arf1, GIT1, Rab23, ARHN, RabGGTA, HIPK2 and CLTC were classified as hits (Figures 1E and Table S1). The identification of IKKα, also known as CHUK, was unexpected as peptidoglycan recognition via Nod1 triggers the canonical NF-κB pathway, and was, therefore, thought to be exclusively dependent on IKKβ and NEMO. The contribution of IKKα in the mechanisms that control NF-κB activation following *S. flexneri* infection was then further explored.

### Depletion of IKKα inhibits *S. flexneri*-induced p65 nuclear translocation and IL-8 expression without affecting bacterial invasion

**Figure 2:**
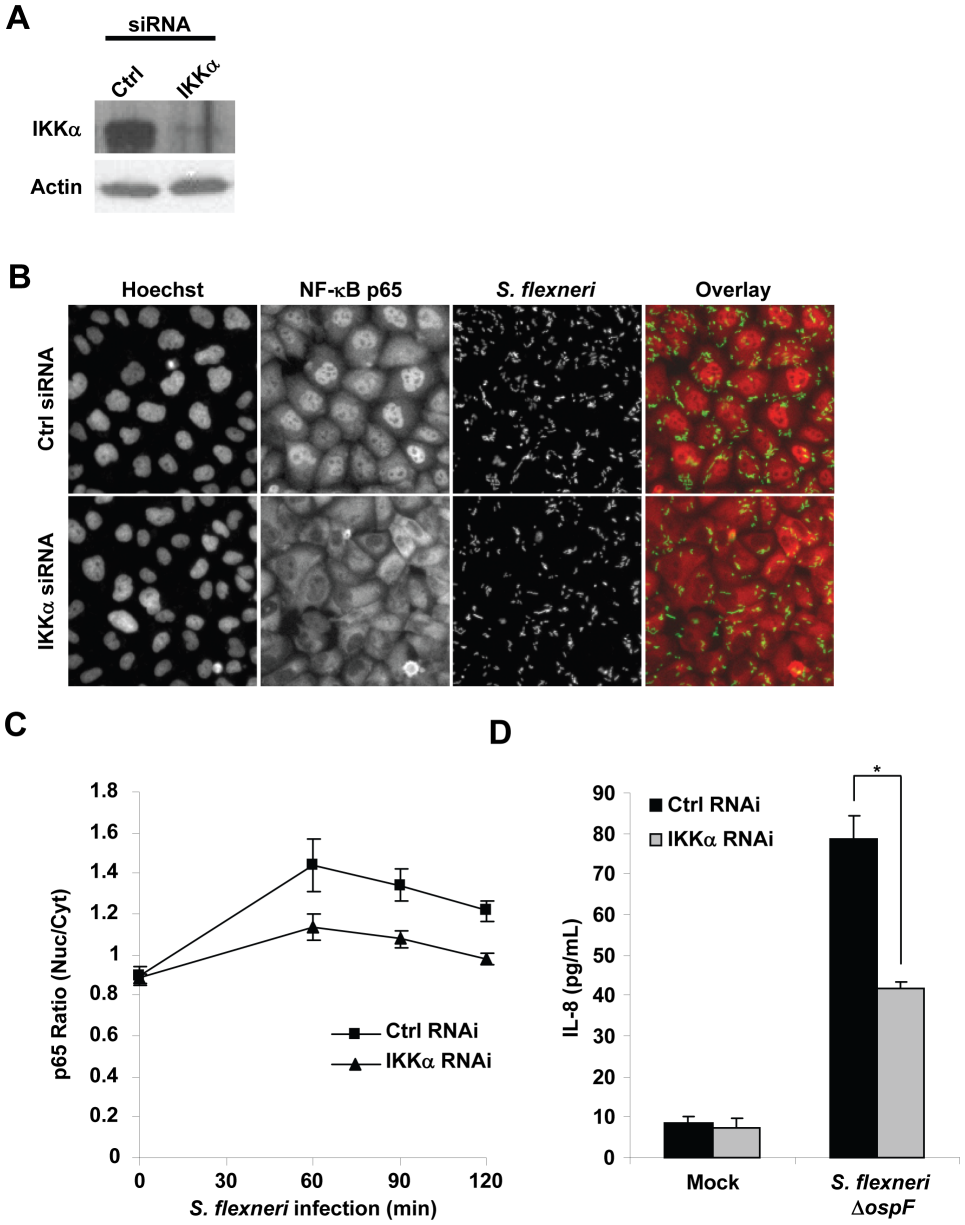
Depletion of IKKα inhibits *S. flexneri*-induced p65 nuclear translocation and IL-8 expression without affecting bacterial invasion. (**A**) IKKα expression is effectively reduced by RNAi. HeLa cells were transfected with the indicated siRNAs. Western blots were performed on cell lysates. Actin levels are shown as a loading control. (**B**) Depletion of IKKα inhibits *S. flexneri*-induced p65 nuclear translocation and IL-8 expression without affecting bacterial invasion. HeLa cells were transfected with control or IKKα siRNA and infected with *S. flexneri* for 60 minutes at MOI = 10. An overlay is shown for *S. flexneri* (green) and p65 (red), right panel. (**C**) Time course of p65 nuclear translocation during *S. flexneri* infection of control and IKKα-depleted cells. Results represent the mean ± SD of 12 images; graph representative of 2 independent experiments. (**D**) *S. flexneri*-induced IL-8 secretion is impaired by the depletion of IKKα. IL-8 secretion was measured by ELISA in the supernatant of control or IKKα-depleted HeLa cells left untreated (Mock) or infected with *S. flexneri* Δ*ospF*. Results represent the mean ± SD of 6 wells; graph representative of 3 independent experiments, *p = 0.006.

First, to validate the role of IKKα in *S. flexneri*-induced p65 nuclear translocation, we tested a pool of four synthetic IKKα siRNAs that had no overlapping sequences with the *in vitro* diced siRNAs used in the screen. In conditions where around 90% of endogenous protein was depleted (Figure 2A), a massive reduction of p65 nuclear translocation was observed (Figures 2B and 2C). This result was not explained by a reduction of bacterial uptake as the number of internalized bacteria was similar in control and in IKKα-depleted cells (Figure 2B). Taken together, these results indicated that IKKα is required for the activation of NF-κB during infection of epithelial cells by *S. flexneri*.

During infection, NF-κB positively regulates the expression of multiple proinflammatory genes [31]. In particular, it induces the expression of the chemokine IL-8, which recruits PMNs on site of infection, and thereby limits the spread of bacterial invasion within the intestinal tissue [18]. To test whether IKKα contributed to the upregulation of IL-8 expression during infection, we measured by ELISA the secretion of IL-8 in the supernatant of control and IKKα-depleted HeLa cells six hours post infection. To increase the amount of IL-8 produced in response to infection, cells were infected with a mutant of *S. flexneri* deleted for the type III effector OspF (Δ*ospF*) that dampens inflammation signaling by dephosphorylating p38 in the nucleus of infected cells [20]. Consistent with NF-κB data, a reduction of IL-8 secretion was observed in response to *S. flexneri* Δ*ospF* infection when cells were depleted for IKKα (Figure 2D), showing that IKKα is involved in the signaling pathways that control the expression of a critical inflammatory chemokine during bacterial infection.

### Both IKKα and IKKβ contribute to the phosphorylation and the degradation of IκBα during *S. flexneri* infection

**Figure 3:**
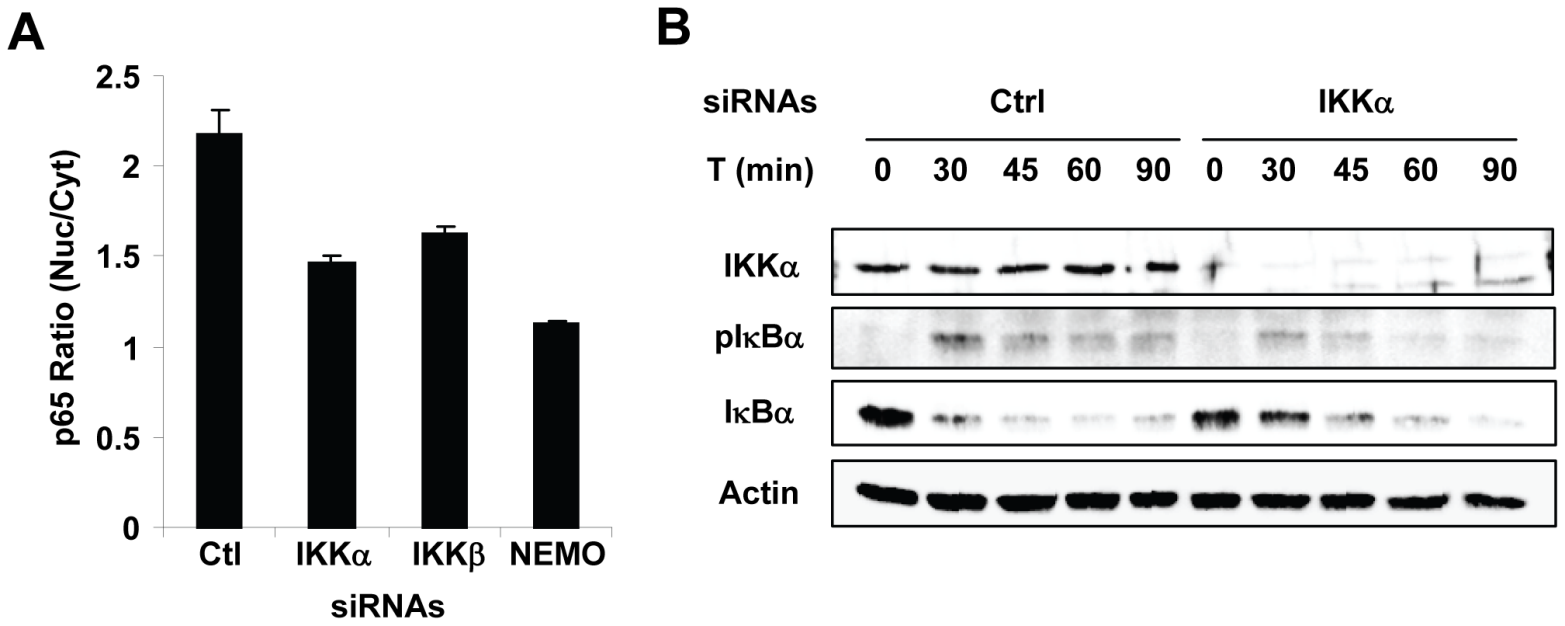
Both IKKα and IKKβ catalytic subunits contribute to NF-κB activation during infection by *S. flexneri*. (**A**) Depletion of IKKα, IKKβ or NEMO reduces the translocation of p65 during *S. flexne*ri. *S. flexneri*-induced p65 translocation was analyzed in HeLa cells transfected with control, IKKα, IKKβ and NEMO siRNAs. Results represent the mean ± SD of 12 images; graph representative of 2 independent experiments. (**B**) IKKα is involved in the phosphorylation and the degradation of IκBα during *S. flexneri* infection. Control or IKKα-depleted HeLa cells were left untreated or infected with *S. flexneri* for the indicated periods at MOI = 10. Levels of IKKα, pIκBα and IκBα were monitored by western blots performed on cell lysates. Actin levels are shown as a loading control.

It is generally believed that IKKβ and NEMO are the two subunits of the IKK complex involved in the canonical pathway of NF-κB activation. To test the contribution of all IKK subunits in the activation of NF-κB during infection of epithelial cells by *S. flexneri*, we monitored the localization of p65 in cells depleted for IKKα, IKKβ or NEMO. As shown in Figure 3A, the depletion of either of these proteins reduced the nuclear translocation of p65, indicating that both catalytic subunits IKKα and IKKβ, as well as the scaffolding function of NEMO were required to fully activate the NF-κB pathway during infection. IKKβ regulates the canonical pathway of NF-κB activation by phosphorylating IκBα at positions serine 32 and 36, thereby inducing its polyubiquitination and subsequent degradation [9]. To test whether IKKα regulated NF-κB via a similar mechanism, we monitored the phosphorylation of IκBα at serines 32 and 36 (pIκBα) and its degradation following infection of HeLa cells by *S. flexneri*. Whereas massive phosphorylation and degradation of IκBα were observed in control cells following infection, these two processes were strongly reduced after IKKα knockdown (Figure 3B), indicating that IKKα largely contributes to the phosphorylation and degradation of IκBα during infection by *S. flexneri*.

### IKKα is involved in Nod1-mediated peptidoglycan recognition

**Figure 4:**
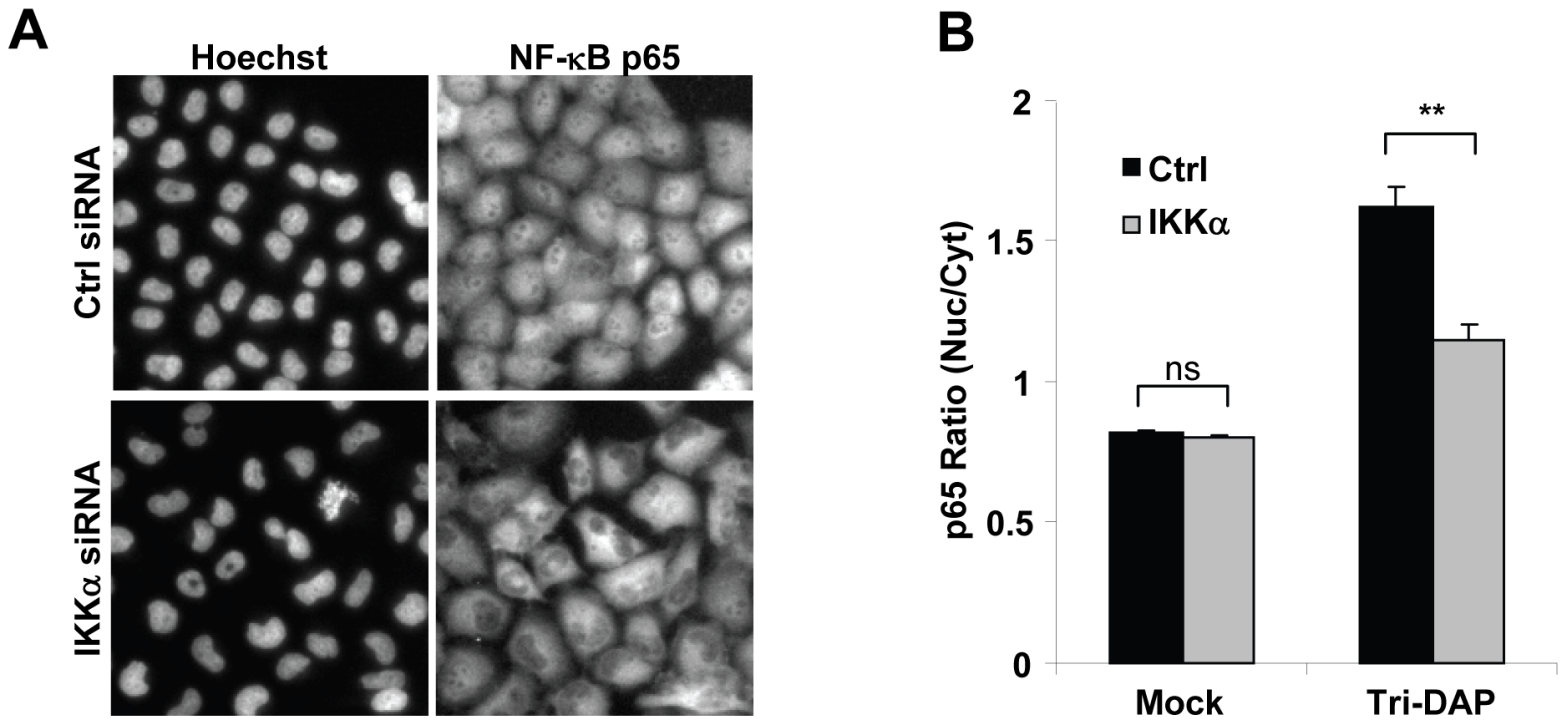
IKKα is required for the activation of NF-κB induced by Nod1-mediated peptidoglycan recognition. (**A**) IKKα depletion reduces the activation of NF-κB induced by Nod1-mediated peptidoglycan recognition. Control or IKKα-depleted HeLa cells were treated with Tri-DAP as described in Materials and Methods. After 1 hour, cells were fixed, stained for p65 and DNA with an anti-p65 antibody and Hoechst, respectively. (**B**) Quantification of the effect of IKKα depletion on Tri-DAP-induced p65 nuclear translocation. Results represent the mean ± SD of 12 images; graph representative of 3 independent experiments, **p≤8.6E-4, ns: non-significant.

During *S. flexneri* infection, the activation of NF-κB is initiated by the recognition of peptidoglycan fragments via the intracellular receptor Nod1 [16]. To specifically examine the implication of IKKα in Nod1-mediated signaling, the effect of IKKα depletion was directly tested in cells exposed to the Nod1 ligand Tri-DAP. Whereas Tri-DAP treatment induced a clear nuclear translocation of p65 in control cells, this translocation was severely impaired in IKKα-depleted cells (Figures 4A and 4B), demonstrating that IKKα is required for the activation of NF-κB following Nod1-mediated peptidoglycan recognition.

## Discussion

Although NF-κB is a transcription factor that has been the subject of intensive research by academic laboratories and the pharmaceutical industry, the complex molecular mechanisms controlling its activation are only partially elucidated. Here we performed an RNAi screen to identify new proteins involved in the mechanisms that control NF-κB activation following pathogen recognition using infection of epithelial cells by the invasive bacterium *S. flexneri* as a model system. The presence of *S. flexneri* in the cytoplasm is recognized via the detection of peptidoglycan by the receptor Nod1 [16]. This recognition leads to NF-κB activation and the upregulation of proinflammatory genes that orchestrate the host inflammatory response [31]. The protein IKKα was identified in the screen as a protein required for the activation of NF-κB in response to *S. flexneri* infection. Since bacterial uptake was not affected by the depletion of IKKα, this result indicated that IKKα is involved in the activation of NF-κB in response to infection. The depletion of IKKβ or NEMO also induced a reduction of *S. flexneri*-induced-p65 nuclear translocation, confirming that all subunits were required for the full activation of NF-κB during infection. As the translocation of p65 depends on the degradation of IκBα primed by its phosphorylation on serines 32 and 36, the role of IKKα in these two processes was analyzed. A reduction of IκBα phosphorylation and delayed degradation were observed in cells depleted for IKKα, indicating that similar to IKKβ, IKKα triggers NF-κB activation by inducing the phosphorylation and the degradation of IκBα. Taken together, these results provided new evidence for a role of IKKα in the canonical pathway of NF-κB activation, and were in line with few reports indicating that the dominant model of canonical NF-κB activation based on IKKβ and NEMO is incomplete. In particular, it has been reported that IKKα is the key subunit responsible for the Receptor activator of NF-κB (RANK)-induced classical NF-κB activation in mammary epithelial cells [32]. In addition, Solt et al. showed that IL-1-induced NF-κB activation requires the interaction of IKKα with NEMO and occurs in the absence of IKKβ [11]. Finally, it has been shown recently that both IKKα and IKKβ contribute to IκBα phosphorylation and NF-κB activation in response to TNFα stimulation in HeLa cells [10]. For the first time, our results demonstrate that IKKα is also implicated in the canonical pathway of NF-κB activation triggered by bacterial infection. To further demonstrate that pathogen recognition induced NF-κB activation in an IKKα-dependent manner during *S. flexneri* infection, we directly tested whether IKKα was involved in the activation of NF-κB induced by Nod1-mediated peptidoglycan recognition. For this purpose, controls or IKKα-depleted cells were directly stimulated with the purified Nod1 ligand Tri-DAP. The analysis of the p65 nuclear translocation showed that IKKα was required for the full activation of NF-κB in response to Tri-DAP treatment, indicating that IKKα is involved in the molecular mechanism signaling the recognition of peptidoglycan-derived peptides by Nod1.

Following infection of epithelial cells by *S. flexneri*, the activation of NF-κB leads to the upregulation of genes encoding for inflammatory cytokines including IL-8 and TNFα [31]. To confirm the implication of IKKα in the expression of genes induced by the canonical NF-κB pathway, the deletion of IKKα was tested on *S. flexneri*-induced IL-8 expression. Consistent with NF-κB data, the secretion of IL-8 was reduced when IKKα was depleted, showing that the contribution of IKKα to NF-κB activation has a functional impact on the amplitude of the inflammatory response mounted in response to bacterial infection. These results suggest that inhibition of IKKα activity may be critical to control inflammation upon bacterial infection. Inhibitors of IKKβ have demonstrated therapeutic benefits in various animal models of inflammatory diseases and are currently in early clinical trials [33], [34], [35]. The data presented in this manuscript suggest that IKKα inhibitors should also be developed and used in combination with IKKβ inhibitors to limit inflammation during bacterial infection or in inflammatory disorders that may involve Nod1 signaling, including asthma, eczema and inflammatory bowel diseases [36], [37], [38].

## Supporting Information

Table S1 Results of the screen on *S. flexneri*‐induced p65 nuclear translocation. Scores are fold standard deviations from the mean of GL3 control p65 ratios. Gene names are based on the NCBI nomenclature. (PDF)
