# Supplementary material for: IKKα Contributes to Canonical NF-κB Activation Downstream of Nod1-Mediated Peptidoglycan Recognition
Source: PLoS One. 2010 Oct 15;5(10):e15371. doi: 10.1371/journal.pone.0015371 (PMC2955547; doi:10.1371/journal.pone.0015371)
Supplement: Table S1 — Results of the screen on S. flexneri‐induced p65 nuclear translocation. Scores are fold standard deviations from the mean of GL3 control p65 ratios. Gene names are based on the NCBI nomenclature. (PDF) [file pone.0015371.s001.pdf]

Table S1. Results of the screen on *S. flexneri*-induced p65 nuclear translocation

Gene	Gene ID	Score	Gene	Gene ID	Score	Gene	Gene ID	Score
CLTC	1213	3.833	RHOB	388	0.260	UBA7	7318	-1.413
ARHGEF7	8874	2.318	IPO7	10527	0.237	HIP1	3092	-1.415
GIPC2	54810	2.009	PTPRO	5800	0.224	PDE7B	27115	-1.428
PRKAG1	5571	1.887	ADCY3	109	0.220	DLG5	9231	-1.464
HIPK3	10114	1.886	DUSP2	1844	0.203	UBE2N	7334	-1.506
DBN1	1627	1.863	HOMER2	9455	-0.056	GRK7	131890	-1.508
HOMER1	9456	1.861	NOD2	64127	-0.125	ARPC3	10094	-1.611
P2RY14	9934	1.845	PRKAG2	51422	-0.144	DGKQ	1609	-1.625
RHOA	387	1.779	AGAP3	116988	-0.210	ADRBK1	156	-1.655
MPP1	4354	1.699	AGAP1	116987	-0.228	SPTB	6710	-1.672
RAC1	5879	1.685	LIN7A	8825	-0.263	FERMT1	55612	-1.717
LIN7C	55327	1.633	DUSP15	128853	-0.297	GUCY2D	3000	-1.751
RAB25	57111	1.626	ADAP1	11033	-0.456	AP2A2	161	-1.799
DUSP6	1848	1.422	RAP1B	5908	-0.509	ARL11	115761	-1.876
NCK2	8440	1.413	ABL1	25	-0.516	CSK	1445	-1.886
EPHB6	2051	1.275	ERN2	10595	-0.545	GRK1	6011	-1.900
ADAP2	55803	1.186	ARPC4	10093	-0.548	ANLN	54443	-1.953
SYK	6850	1.184	GUCY1A2	2977	-0.571	TAB1	10454	-2.043
ERN1	2081	1.146	RAP1GAP	5909	-0.603	GRLF1	2909	-2.060
CAND1	55832	1.138	CCNYL1	151195	-0.605	ARF6	382	-2.136
RAP1A	5906	1.125	GUCA1B	2979	-0.645	HIPK4	147746	-2.224
MTMR4	9110	1.046	ACAP1	9744	-0.678	ZAP70	7535	-2.252
RAC2	5880	1.045	PTEN	5728	-0.698	AP1B1	162	-2.305
PPP2R5E	5529	1.019	DGKG	1608	-0.730	PHIP	55023	-2.366
MTMR2	8898	0.990	HIPK1	204851	-0.739	RAB14	51552	-2.390
ARHGEF9	23229	0.978	ARHGAP25	9938	-0.763	ACAP1	9744	-2.435
NCK1	4690	0.963	MTMR3	8897	-0.855	NUAK1	9891	-2.524
HOMER3	9454	0.912	GIT2	9815	-0.949	LAT	27040	-2.575
SNRK	54861	0.885	AGAP2	116986	-0.950	LIN7B	64130	-2.628
AP2B1	163	0.871	RAC3	5881	-0.954	TNK2	10188	-2.974
AAK1	22848	0.819	MTMR8	55613	-0.958	RGS19	10287	-3.398
DUSP16	80824	0.765	ADCY8	114	-0.989	PTPN14	5784	-3.416
DGKA	1606	0.677	GRK6	2870	-0.990	HIPK2	28996	-3.523
ACAP2	23527	0.675	PTPN18	26469	-0.991	RABGGTA	5875	-3.567
GRK4	2868	0.653	ARL1	400	-1.022	RND2	8153	-3.801
CD2AP	23607	0.616	UBE3A	7337	-1.028	RAB23	51715	-3.979
UBE2D1	7321	0.534	ABI1	10006	-1.061	KIF11	3832	-4.053
EPHA3	2042	0.523	EPS8	2059	-1.062	GIT1	28964	-4.124
RAP2C	57826	0.473	BUB1B	701	-1.132	ARF1	375	-4.664
SHROOM3	57619	0.462	GUCA1B	2979	-1.201	FGD3	89846	-4.722
ARRB2	409	0.401	PRKACB	5567	-1.204	SRC	6714	-5.509
APPL1	26060	0.354	ACAP3	116983	-1.244	CHUK	1147	-6.098
ARRB1	408	0.332	GRK5	2869	-1.281	RIPK2	8767	-7.127
EPHA5	2044	0.291	UBA6	55236	-1.302	NOD1	10392	-7.951

Scores are fold standard deviations from the mean of GL3 control p65 ratios. Gene names are based on the NCBI nomenclature.
